# Influence of Landau level mixing on the properties of elementary excitations in graphene in strong magnetic field

**DOI:** 10.1186/1556-276X-7-134

**Published:** 2012-02-16

**Authors:** Yurii E Lozovik, Alexey A Sokolik

**Affiliations:** 1 Institute for Spectroscopy, Russian Academy of Sciences, Fizicheskaya 5, 142190, Troitsk, Moscow Region, Russia; 2 Moscow Institute of Physics and Technology, Institutskii Per. 9, 141700, Dolgoprudny, Moscow Region, Russia

## Abstract

Massless Dirac electrons in graphene fill Landau levels with energies scaled as square roots of their numbers. Coulomb interaction between electrons leads to mixing of different Landau levels. The relative strength of this interaction depends only on dielectric susceptibility of surrounding medium and can be large in suspended graphene. We consider influence of Landau level mixing on the properties of magnetoexcitons and magnetoplasmons—elementary electron-hole excitations in graphene in quantizing magnetic field. We show that, at small enough background dielectric screening, the mixing leads to very essential change of magnetoexciton and magnetoplasmon dispersion laws in comparison with the lowest Landau level approximation.

PACS: 73.22.Pr; 71.35.Ji; 73.43.Mp; 71.70.Gm.

## 1 Introduction

Two-dimensional systems in strong magnetic field are studied intensively since the discovery of integer and fractional quantum Hall effects [1-3]. For a long time, such systems were represented by gallium arsenide heterostructures with 2D electron motion within each subband [4].

New and very interesting realization of 2D electron system appeared when graphene, a monoatomic layer of carbon, was successfully isolated [5,6]. The most spectacular property of graphene is the fact that its electrons behave as massless chiral particles, obeying Dirac equation. Intensive experimental and theoretical studies of this material over several recent years yielded a plethora of interesting results [7-9]. In particular, graphene demonstrates unusual half-integer quantum Hall effect [6], which can be observed even at room temperature [10].

In external perpendicular magnetic field, the motion of electrons along cyclotron orbits acquires zero-dimensional character and, as a result, electrons fill discrete Landau levels [11]. In semiconductor quantum wells, Landau levels are equidistant and separation between them is determined by the cyclotron frequency *ω*_c _= *eH*/*mc*. In graphene, due to massless nature of electrons, "ultra-relativistic" Landau levels appear, which are non-equidistant and located symmetrically astride the Dirac point [12,13]. Energies of these levels are $E_{n}=\text{sign}\left(n\right)\sqrt{2\left|n\right|}v_{F}/l_{H}$, where *n *= 0, ±1, ±2,...,* v*_*F *_≈10^6 ^m/s is the Fermi velocity of electrons and $l_{H}=\sqrt{c/eH}$ is magnetic length, or radius of the cyclotron orbit (here and below we assume *ħ *= 1).

In the case of integer filling, when several Landau levels are completely filled by electrons and all higher levels are empty, elementary excitations in the system are caused by electron transitions from one of the filled Landau levels to one of the empty levels [14]. Such transitions can be observed in cyclotron resonance or Raman scattering experiments as absorption peaks at certain energies. With neglect of Coulomb interaction, energy of the excited electron-hole pair is just a distance between Landau levels of electron and hole. Coulomb interaction leads to mixing of transitions between different pairs of Landau levels, changing the resulting energies of elementary excitations.

Characteristic energy of Coulomb interaction in magnetic field is *e*^2^/*εl*_*H*_, where *ε *is a dielectric permittivity of surrounding medium. The relative strength of Coulomb interaction can be estimated as ratio of its characteristic value to a characteristic distance between Landau levels. For massive electrons in semiconductor quantum wells, this ratio is proportional to *H*^-1/2^, thus in asymptotically strong magnetic field the Coulomb interaction becomes a weak perturbation [15,16]. In this case, the lowest Landau level approximation, neglecting Landau level mixing, is often used. It was shown that Bose-condensate of noninteracting magnetoexcitons in the lowest Landau level is an exact ground state in semiconductor quantum well in strong magnetic field [17].

A different situation arises in graphene. The relative strength of Coulomb interaction in this system can be expressed as *r*_s _= *e*^2^/*εv*_*F *_and does not depend on magnetic field [18]. The only parameter which can influence it is the dielectric permittivity of surrounding medium *ε*. At small enough *ε*, mixing between different Landau levels can significantly change properties of elementary excitations in graphene.

Coulomb interaction leads to appearance of two types of elementary excitations from the filled Landau levels. From summation of "ladder" diagrams we get magnetoexcitons, which can be imagined as bound states of electron and hole in magnetic field [14,16,19]. Properties of magnetoexcitons in graphene were considered in several works, mainly in the lowest Landau level approximation [20-24]. At *ε *≈ 3, Landau level mixing was shown to be weak in the works [20,25].

Note that influence of Landau level mixing on properties of an insulating ground state of neutral graphene was considered in [26] by means of tight-binding Hartree-Fock approximation. It was shown that Landau level mixing favors formation of insulating charge-density wave state instead of ferromagnetic and spin-density wave states in suspended graphene, i.e., at weak enough background dielectric screening.

From the experimental point of view, the most interesting are magnetoexcitons with zero total momentum, which are only able to couple with electromagnetic radiation due to very small photon momentum. For usual non-relativistic electrons, magnetoexciton energy at zero momentum is protected against corrections due to electron interactions by the Kohn theorem [27]. However, for electrons with linear dispersion in graphene the Kohn theorem is not applicable [21,24,28-32]. Thus, observable energies of excitonic spectral lines can be seriously renormalized relatively to the bare values, calculated without taking into account Coulomb interaction.

The other type of excitations can be derived using the random phase approximation, corresponding to summation of "bubble" diagrams. These excitations, called magnetoplasmons, are analog of plasmons and have been studied both in 2D electron gas [14,33] and graphene [18,20,21,24,34-39] (both with and without taking into account Landau level mixing).

In the present article, we consider magnetoexcitons and magnetoplasmons with taking into account Landau level mixing and show how the properties of these excitations change in comparison with the lowest Landau level approximation. For magnetoexcitons, we take into account the mixing of asymptotically large number of Landau levels and find the limiting values of cyclotron resonance energies.

For simplicity and in order to stress the role of virtual transitions between different pairs of electron and hole Landau levels (i.e., the role of two-particle processes), here we do not take into account renormalization of single-particle energies via exchange with the filled levels. This issue have been considered in several theoretical studies [20,21,24,30,40]. Correction of Landau level energies can be treated as renormalization of the Fermi velocity, dependent on the ultraviolet cutoff for a number of the filled Landau levels taken into account in exchange processes.

The rest of this article is organized as follows. In Section 2, we present a formalism for description of magnetoexcitons in graphene, which is applied in Section 3 to study influence of Coulomb interaction and Landau level mixing on their properties. In Section 4, we study magnetoplasmons in graphene in the random phase approximation and in Section 5 we formulate the conclusions.

## 2 Magnetoexcitons

Electrons in graphene populate vicinities of two nonequivalent Dirac points in the Brillouin zone, or two valleys **K **and **K'**. We do not consider intervalley scattering and neglect valley splitting, thus it is sufficient to consider electrons in only one valley and treat existence of the other valley as additional twofold degeneracy of electron states.

We consider magnetoexciton as an electron-hole pair, and we will denote all electron and hole variables by the indices 1 and 2 respectively. In the valley **K**, Hamiltonian of free electrons in graphene in the basis {*A*_1_,*B*_1_} of sublattices takes a form [7]:

(1) $$H_{1}^{\left(0\right)}=v_{F}\sqrt{2}\left(\begin{array}{cc} 0 & p_{1-}\\ p_{1+} & 0 \end{array}\right),$$

where $p_{1\pm }=\left(p_{1x}\pm ip_{1y}\right)/\sqrt{2}$ are the cyclic components of electron momentum and *v*_*F *_≈ 10^6^m/s is the Fermi velocity of electrons.

For external magnetic field **H**, parallel to the *z *axis, we take the symmetrical gauge, when $A\left(r\right)=\frac{1}{2}\left[H\times r\right]$. Introducing the magnetic field as substitution of the momentum **p**_1 _→ **p**_1 _+ (*e*/*c*)**A**(**r**_1_) in (1) (we treat the electron charge as -*e*), we get the Hamiltonian of the form:

(2) $$H_{1}=\frac{v_{\text{F}}\sqrt{2}}{l_{H}}\left(\begin{array}{cc} 0 & a_{1}\\ a_{1}^{+} & 0 \end{array}\right).$$

Here the operators $a_{1}=l_{HP1-}-ir_{1-}/2l_{H}$ and $a_{1}^{+}=l_{HP1+}+ir_{1+}/2l_{H}$ (where $r_{1\pm }=\left(x_{1}\pm iy_{1}\right)/\sqrt{2}$) obey bosonic commutation relation $\left[a_{1},a_{1}^{+}\right]=1$.

Using this relation, by means of successive action of the raising operator $a_{1}^{+}$ we can construct Landau levels for electron [18] with energies

(3) $$E_{n}^{\text{L}}=s_{n}\sqrt{2\left|n\right|}\frac{v_{\text{F}}}{l_{H}}$$

and wave functions

(4) $$\psi_{nk}\left(r\right)={\left(\sqrt{2}\right)}^{\delta_{n0}-1}\left(\begin{array}{c} s_{n}\phi_{\left|n\right|-1,k}\left(r\right)\\ \phi_{\left|n\right|k}\left(r\right) \end{array}\right).$$

Here *k *= 0,1, 2,... is the index of guiding center, which enumerates electron states on the *n*th Landau level (*n *= -∞,...,+∞), having macroscopically large degeneracy $N_{\phi }=S/2\pi l_{H}^{2}$, equal to a number of magnetic flux quanta penetrating the system of the area *S*. Eigenfunctions *ϕ*_*nk*_(**r**) of a 2D harmonic oscillator have the explicit form:

(5) $$\begin{array}{c} \phi_{nk}\left(r\right)=\frac{i^{\left|n-k\right|}}{\sqrt{2\pi }l_{H}}\sqrt{\frac{\text{min}\left(n,k\right)!}{\text{max}\left(n,k\right)!}}e^{-r^{2}/4l_{H}^{2}}\\ \times {\left(\frac{x+is_{n-k}y}{\sqrt{2}l_{H}}\right)}^{\left|n-k\right|}L_{\text{min}\left(n,k\right)}^{\left|n-k\right|}\left(\frac{r^{2}}{2l_{H}^{2}}\right), \end{array}$$

*s*_*n *_= sign(*n*) and $L_{n}^{\alpha }\left(x\right)$ are associated Laguerre polynomials.

Consider now the hole states. A hole wave function is a complex conjugated electron wave function, and the hole charge is +*e*. Thus, we can obtain Hamiltonian of the hole in magnetic field from the electron Hamiltonian (2) by complex conjugation and reversal of the sign of the vector potential **A**(**r**_2_). In the representation of sublattices {*A*_2_,*B*_2_} it is

(6) $$H_{2}=\frac{v_{\text{F}}\sqrt{2}}{l_{H}}\left(\begin{array}{cc} 0 & a_{2}\\ a_{2}^{+} & 0 \end{array}\right),$$

where the operators $a_{2}=l_{HP2+}-ir_{2+}/2l_{H}$ and $a_{2}^{+}=l_{HP2-}+ir_{2-}/2l_{H}$ commute with $a_{1},a_{1}^{+}$ and obey the commutation relation $\left[a_{2},a_{2}^{+}\right]=1$. Energies of the hole Landau levels are the same as these of electron Landau levels (3), but have an opposite sign.

Hamiltonian of electron-hole pair without taking into account Landau level mixing is just the sum of (2) and (6), and can be represented in the combined basis of electron and hole sublattices {*A*_1_*A*_2_,*A*_1_*B*_2_,*B*_1_*A*_2_,*B*_1_*B*_2_} as

(7) $$H_{0}=H_{1}+H_{2}=\frac{v_{\text{F}}\sqrt{2}}{l_{H}}\left(\begin{array}{cccc} 0 & a_{2} & a_{1} & 0\\ a_{2}^{+} & 0 & 0 & a_{1}\\ a_{1}^{+} & 0 & 0 & a_{2}\\ 0 & a_{1}^{+} & a_{2}^{+} & 0 \end{array}\right).$$

It is known [41] that for electron-hole pair in magnetic field there exists a conserving 2D vector of magnetic momentum, equal in our gauge to

(8) $$P=p_{1}+p_{2}-\frac{e}{2c}\left[H\times \left(r_{1}-r_{2}\right)\right]$$

and playing the role of a center-of-mass momentum. The magnetic momentum is a generator of simultaneous translation in space and gauge transformation, preserving invariance of Hamiltonian of charged particles in magnetic field [42].

The magnetic momentum commutes with both the noninteracting Hamiltonian (7) and electron-hole Coulomb interaction *V*(**r**_1_-**r**_2_). Therefore, we can find a wave function of magnetoexciton as an eigenfunction of (8):

(9) $$\begin{array}{c} \Psi_{Pn_{1}n_{2}}\left(r_{1},r_{2}\right)=\frac{1}{2\pi }\text{exp}\left\{iR\left(P+\frac{\left[e_{z}\times r\right]}{2l_{H}^{2}}\right)\right\}\\ \;\;\;\;\;\;\;\;\;\;\;\times \Phi_{n_{1}n_{2}}\left(r-r_{0}\right). \end{array}$$

Here **R **= (**r**_1 _+ **r**_2_)*/*2, **r **= **r**_1 _- **r**_2_, **e**_*z *_is a unit vector in the direction of the *z *axis. The wave function of relative motion of electron and hole $\Phi_{n_{1}n_{2}}\left(r-r_{0}\right)$ is shifted on the vector $r_{0}=l_{H}^{2}\left[e_{z}\times P\right]$. This shift can be attributed to electric field, appearing in the moving reference frame of magnetoexciton and pulling apart electron and hole.

Transformation (9) from **Ψ **to **Φ **can be considered as a unitary transformation **Φ **= *U***Ψ**, corresponding to a switching from the laboratory reference frame to the magnetoexciton rest frame. Accordingly, we should transform operators as *A *→ *UAU*^+^. Transforming the operators in (7), we get: $Ua_{1}U^{+}=b_{1},Ua_{1}^{+}U^{+}=b_{1}^{+},Ua_{2}U^{+}=-b_{2},Ub_{2}^{+}U^{+}=-b_{2}^{+}$. Here the operators $b_{1}=l_{HP-}-ir_{-}/2l_{H},b_{1}^{+}=l_{HP+}+ir_{+}/2l_{H},b_{2}=l_{HP+}-ir_{+}/2l_{H},b_{2}^{+}=l_{HP-}+ir_{-}/2l_{H}$ contain only the relative electron-hole coordinate and momentum and obey commutation relations $\left[b_{1},b_{1}^{+}\right]=1,\left[b_{2},b_{2}^{+}\right]=1$ (all other commutators vanish).

Thus, the Hamiltonian (7) of electron-hole pair in its center-of-mass reference frame takes the form

(10) $$H_{0}^{\prime }=\frac{v_{\text{F}}\sqrt{2}}{l_{H}}\left(\begin{array}{cccc} 0 & -b_{2} & b_{1} & 0\\ -b_{2}^{+} & 0 & 0 & b_{1}\\ b_{1}^{+} & 0 & 0 & -b_{2}\\ 0 & b_{1}^{+} & -b_{2}^{+} & 0 \end{array}\right).$$

A four-component wave function of electron-hole relative motion $\Phi_{n_{1}n_{2}}$, being an eigenfunction of (10), can be constructed by successive action of the raising operators $b_{1}^{+}$ and $b_{2}^{+}$ (see also [20,21]):

(11) $$\begin{array}{c} \Phi_{n_{1}n_{2}}\left(r\right)={\left(\sqrt{2}\right)}^{\delta_{n_{1},0}+\delta_{n_{2},0}-2}\\ \times \left(\begin{array}{c} s_{n_{1}}s_{n_{2}}\phi_{\left|n_{1}\right|-1,\left|n_{2}\right|-1}\left(r\right)\\ s_{n_{1}}\phi_{\left|n_{1}\right|-1,\left|n_{2}\right|}\left(r\right)\\ s_{n_{2}}\phi_{\left|n_{1}\right|,\left|n_{2}\right|-1}\left(r\right)\\ \phi_{\left|n_{1}\right|\left|n_{2}\right|}\left(r\right) \end{array}\right). \end{array}$$

The bare energy of magnetoexciton in this state is a difference between energies (3) of electron and hole Landau levels:

(12) $$E_{n_{1}n_{2}}^{\left(0\right)}=E_{n_{1}}^{L}-E_{n_{2}}^{L}.$$

Here we label the state of relative motion by numbers of Landau levels *n *_1 _and *n*_2 _of electron and hole, respectively. The whole wave function of magnetoexciton (9) is additionally labeled by the magnetic momentum **P**. In the case of integer filling, when all Landau levels up to *ν*th one are completely filled by electrons and all upper levels are empty, magnetoexciton states with *n*_1 _>*ν, n*_2 _≤ *ν *are possible. For simplicity, we neglect Zeeman and valley splittings of electron states, leading to appearance of additional spin-flip and intervalley excitations [20,21,24].

## 3 Influence of Coulomb interaction

Now we take into account the Coulomb interaction between electron and hole *V*(**r**) = -*e*^2^/*εr*, screened by surrounding dielectric medium with permittivity *ε*. Upon switching into the electron-hole center-of-mass reference frame, it is transformed as *V*'(**r**) = *V*(**r **+ **r**_0_). To obtain magnetoexciton energies with taking into account Coulomb interaction, we should find eigenvalues of the full Hamiltonian of relative motion $H^{\prime }=H_{0}^{\prime }+V^{\prime }$ in the basis of the bare magnetoexcitonic states (11). As discussed in the Introduction, a relative strength of the Coulomb interaction is described by the dimensionless parameter

(13) $$r_{s}=\frac{e^{2}}{\varepsilon v_{\text{F}}}\approx \frac{2.2}{\varepsilon }.$$

When *ε *>> 1, *r*_s _<< 1 and we can treat Coulomb interaction as a weak perturbation and calculate magnetoexciton energy in the first order in the interaction as:

(14) $$E_{n_{1}n_{2}}^{\left(1\right)}\left(P\right)=E_{n_{1}n_{2}}^{\left(0\right)}+\left\langle \Phi_{n_{1}n_{2}}\left|V^{\prime }\right|\Phi_{n_{1}n_{2}}\right\rangle .$$

Due to spinor nature of electron wave functions in graphene, the correction (14) to the bare magnetoexciton energy (12) is a sum of four terms, each of them having a form of correction to magnetoexciton energy in usual 2D electron gas [20-22]. Dependence of magnetoexciton energy on magnetic momentum **P **can be attributed to Coulomb interaction between electron and hole, separated by the average distance *r*_0 _~ *P*.

Calculations of magnetoexciton dispersions in the first order in Coulomb interaction (14) have been performed in several studies [20-24]. However, such calculations are well-justified only at small enough *r*_s_, i.e., at large *ε*. When *ε *~ 1 (this is achievable in experiments with suspended graphene [43-46]), the role of virtual electron transitions between different Landau levels can be significant.

To take into account Landau level mixing, we should perform diagonalization of full Hamiltonian of Coulomb interacting electrons in some basis of magnetoexcitonic states $\Psi_{Pn_{1}n_{2}}$, where electron Landau levels *n*_1 _>*ν *are unoccupied and hole Landau levels *n*_2 _≤ *ν *are occupied. To obtain eigenvalues of the Hamiltonian, we need to solve the equation:

(15) $$\det\left\|\delta_{{n^{\prime }}_{1}n_{1}}\delta_{{n^{\prime }}_{2}n_{2}}(E_{n_{1}n_{2}}^{\left(0\right)}-E)+\langle \Psi_{P{n^{\prime }}_{1}{n^{\prime }}_{2}}|V|\Psi_{P{n^{\prime }}_{1}{n^{\prime }}_{2}}\rangle \right\|=0.$$

We can constrain our basis to *N*^2 ^terms, involving *N *Landau levels for electron (*n*_1 _= *ν *+ 1,..., *ν *+ *N*) and *N *Landau levels for a hole (*n*_2 _= *ν*,..., *ν *- *N *+ 1). Since the Hamiltonian commutes with magnetic momentum **P**, the procedure of diagonalization can be performed independently at different values of **P**, resulting in dispersions $E_{n_{1}n_{2}}^{\left(N\right)}\left(P\right)$ of magnetoexcitons, affected by a mixing between *N *electron and *N *hole Landau levels.

**Figure 1:**
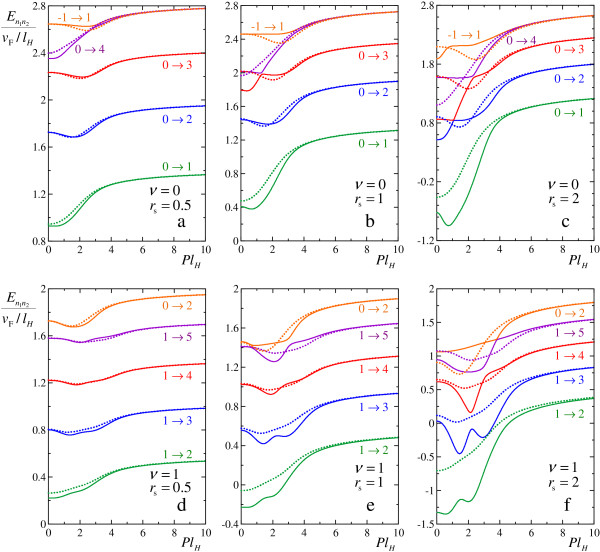
**Magnetoexciton dispersions**. Magnetoexciton dispersions $E_{n_{1}n_{2}}\left(P\right)$, calculated in the first order in Coulomb interaction (dotted lines) and with taking into account mixing between 16 low-lying magnetoexciton states (solid lines). The dispersions are calculated at different filling factors *ν *and different *r*_s_: (**a**) *ν *= 0, *r*_s _= 0.5, (**b**) *ν *= 0, *r*_s _= 1, (**c**) *ν *= 0, *r*_s _= 2, (**d**) *ν *= 1, *r*_s _= 0.5, (**e**) *ν *= 1, *r*_s _= 1, (**f**) *ν *= 1, *r*_s _= 2. Dispersions of 5 lowest-lying magnetoexciton states *n*_2 _→ *n*_1 _indicated near the corresponding curves, are shown.

We present in Figure 1 dispersion relations for 5 lowest magnetoexciton states, calculated with and without taking into account the mixing between 16 lowest-energy states. The results are shown for Landau level fillings *ν *= 0 and *ν *= 1, and for different values of *r*_s_. Close to *P *= 0, magnetoexciton can be described as a composite particle with parabolic dispersion, characterized by some effective mass $M_{n_{1},n_{2}}={\left[d^{2}E_{n_{1},n_{2}}\left(P\right)/dP^{2}\right]}^{-1}{|}_{P=0}$. At large *P*, the Coulomb interaction weakens and the dispersions tend to the energies of one-particle excitations (12). However, the dispersion can have rather complicated structure with several minima and maxima at intermediate momenta $P\sim l_{H}^{-1}$.

We see that the mixing at small *r*_s _has a weak effect on the dispersions (solid and dotted lines are very close in Figure 1a,d). However, at *r*_s _~ 1 the mixing changes the dispersions significantly. We can observe avoided crossings between dispersions of different magnetoexcitons, and even reversal of a sign of magnetoexciton effective masses (see Figure 1b,c,e,f). Also we see that the high levels are more strongly mixed than the low-lying ones. Similar results were presented in [20] for *r*_s _= 0.73 with conclusion that the mixing is weak.

As we see, at large *r*_s _the mixing of several Landau levels already strongly changes magnetoexciton dispersions. Important question arises here: how many Landau levels should we take into account to achieve convergency of results? To answer this question, we perform diagonalization of the type (15), increasing step-by-step a quantity *N *of electron and hole Landau levels. For simplicity, we perform these calculations at *P *= 0 only. Energies of magnetoexcitons at rest, renormalized by electron interactions due to breakdown of the Kohn theorem, are the most suitable to be observed in optical experiments.

**Figure 2:**
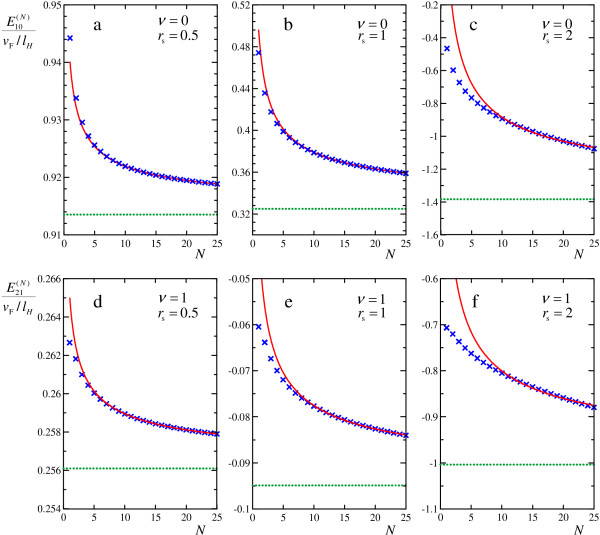
**Magnetoexciton energies with Landau level mixing**. Magnetoexciton energies at rest $E_{n_{1}n_{2}}^{\left(N\right)}\left(P=0\right)$, calculated with taking into account *N *electron and *N *hole Landau levels, with stepwise increasing *N *(crosses). The fits to these energies with inverse-square-root function (solid lines) and limiting values of $E_{n_{1}n_{2}}^{\left(N\right)}\left(P=0\right)$ at *N *→ ∞ (dotted lines) are also shown. The results are presented for different filling factors *ν *and different *r*_s_: **(a) ***ν *= 0, *r*_s _= 0.5, **(b) ***ν *= 0, *r*_s _= 1, **(c) ***ν *= 0, *r*_s _= 2, **(d) ***ν *= 1, *r*_s _= 0.5, (e) *ν *= 1, *r*_s _= 1, **(f) ***ν *= 1, *r*_s _= 2.

The results of such calculations of $E_{n_{1}n_{2}}^{\left(N\right)}\left(P=0\right)$ as functions of *N *are shown in Figure 2 by cross points. We found semi-analytically that eigenvalues of the Hamiltonian under consideration should approach a dependence

(16) $$E_{n_{1}n_{2}}^{\left(N\right)}\approx E_{n_{1}n_{2}}^{\left(\infty \right)}+\frac{C_{n_{1}n_{2}}}{\sqrt{N}}$$

at large *N*. We fitted the numerical results by this dependence and thus were able to find the limiting values $E_{n_{1}n_{2}}^{\left(\infty \right)}$ of magnetoexciton energies with infinite number of Landau levels taken into account.

We see in Figure 2 that the differences between magnetoexciton energies calculated in the first order in Coulomb interaction (the crosses at *N *= 1) and the energies calculated with taking into account mixing between all Landau levels (dotted lines) are very small at *r*_s _= 0.5 (Figure 2a,b), moderate at *r*_s _= 1 (Figure 2b,e) and very large at *r*_s _= 2 (Figure 2c,f). Since convergency of the inverse-square-root function is very slow, even the mixing of rather large (of the order of tens) number of Landau levels is not sufficient to obtain reliable results for magnetoexciton energies, as clearly seen in the Figure 2.

Note that the mixing increases magnetoexciton binding energies, similarly to results on magnetoexcitons in semiconductor quantum wells [47,48].

## 4 Magnetoplasmons

Magnetoplasmons are collective excitations of electron gas in magnetic field, occurring as poles of density-to-density response function. In the random phase approximation, dispersion of magnetoplasmon is determined as a root of the equation

(17) 1-V(q)Π(q,ω)=0,

where *V*(*q*) = 2*πe*^2^/*εq *is the 2D Fourier transform of Coulomb interaction and Π(*q,ω*) is a polarization operator (or polarizability). Polarization operator for graphene in magnetic field can be expressed using magnetoexciton wave functions (11) and energies (12) (see also, [18,32,34-38]):

(18) $$\Pi \left(q,\omega \right)=g\underset{n_{1}n_{2}}{\sum }\frac{f_{n_{2}}-f_{n_{1}}}{\omega -E_{n_{1}n_{2}}^{\left(0\right)}-i\delta }F_{n_{1}n_{2}}\left(q\right),$$

(19) $$\begin{array}{c} \;F_{n_{1}n_{2}}\left(q\right)=\Phi_{n_{1}n_{2}}^{+}\left(ql_{H}^{2}\right)\\ \times \left(\begin{array}{cccc} 1 & 0 & 0 & 1\\ 0 & 0 & 0 & 0\\ 0 & 0 & 0 & 0\\ 1 & 0 & 0 & 1 \end{array}\right)\Phi_{n_{1}n_{2}}\left(ql_{H}^{2}\right), \end{array}$$

where *g *= 4 is the degeneracy factor and *f*_*n *_is the occupation number for the *n*th Landau level, i.e., *f*_*n *_= 1 at *n *≤ *ν *and *f*_*n *_= 0 at *n *>*ν *(we neglect temperature effects since typical separation between Landau levels in graphene in quantizing magnetic field is of the order of room temperature [10]). The matrix between magnetoexcitonic wave functions in (19) ensures that electron and hole belong to the same sublattice, that is needed for Coulomb interaction in exchange channel treated as annihilation of electron and hole in one point of space and subsequent creation of electron-hole pair in another point.

Unlike electron gas without magnetic field, having a single plasmon branch, Equations (17)-(19) give an infinite number of solutions $\omega =\Omega_{n_{1}n_{2}}\left(q\right)$, each of them can be attributed to specific inter-Landau level transition *n*_2 _→ *n*_1 _affected by Coulomb interaction [18,37,38]. Note that at *q *→ 0, when Coulomb interaction *V*(*q*) becomes weak, dispersion of each magnetoplasmon branch $\Omega_{n_{1}n_{2}}\left(q\right)$ tends to the corresponding single-particle excitation energy $E_{n_{1}n_{2}}^{\left(0\right)}$.

At *r*_s _<< 1, we can suppose that magnetoplasmon energy $\Omega_{n_{1}n_{2}}\left(q\right)$ does not differ significantly from the single-particle energy $E_{n_{1}n_{2}}^{\left(0\right)}$. In this case a dominant contribution to the sum in (18) comes from the term with the given *n*_1 _and *n*_2_. Neglecting all other terms, we can write (18) as

(20) $$\Pi \left(q,\omega \right)\approx g\frac{F_{n_{1}n_{2}}\left(q\right)}{\omega -E_{n_{1}n_{2}}^{\left(0\right)}-i\delta },$$

and from (17) we obtain an approximation to plasmon dispersion in the first order in the Coulomb interaction:

(21) $$\Omega_{n_{1}n_{2}}\left(q\right)\approx E_{n_{1}n_{2}}^{\left(0\right)}+gV\left(q\right)F_{n_{1}n_{2}}\left(q\right).$$

Magnetoplasmons in graphene were considered without taking into account Landau level mixing in a manner of Equation (21) in the studies [20,39]. Other authors [21,24,34] took into account several Landau levels, and the others [35-38] performed full summation in the framework of the random phase approximation (17)-(19) to calculate magnetoplasmon dispersions.

**Figure 3:**
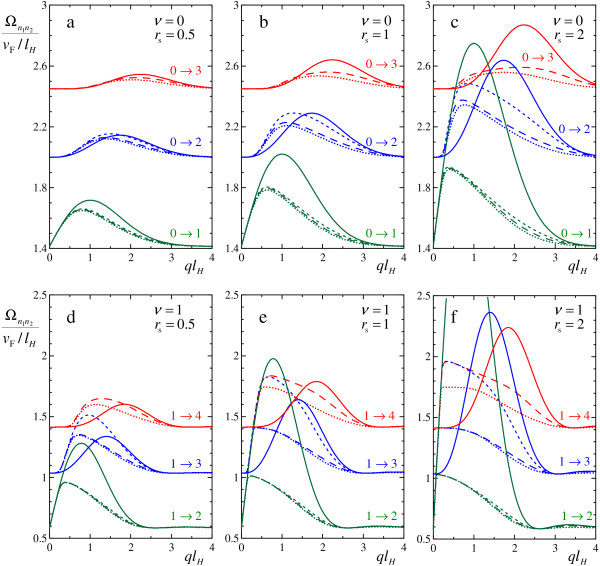
**Magnetoplasmon dispersions**. Magnetoplasmon energies $\Omega_{n_{1}n_{2}}$, calculated in the lowest Landau level approximation (solid lines), with taking into account mixing between 2 (short dash lines) and 3 (long dash lines) Landau levels of electron and hole, and with taking into account mixing between all Landau levels (dotted lines). The results are presented for different filling factors *ν *and different *r*_s_: **(a) ***ν *= 0, *r*_s _= 0.5, **(b) ***ν *= 0, *r*_s _= 1, **(c) ***ν *= 0, *r*_s _= 2, **(d) ***ν *= 1, *r*_s _= 0.5, **(e) ***ν *= 1, *r*_s _= 1, **(f) ***ν *= 1, *r*_s _= 2. Dispersions of 3 lowest-lying magnetoplasmon modes *n*_2 _→ *n*_1_, indicated near the corresponding curves, are shown.

Here we state the question: how many Landau levels one should take into account to calculate magnetoplasmon spectrum with sufficient accuracy? To answer it, we performed calculations with successive taking into account increasing number of Landau levels at different *ν *and *r*_s_. In Figure 3, dispersions of magnetoplasmons in graphene calculated numerically are shown. Results obtained without taking into account Landau level mixing, with taking into account a mixing of two or three lowest Landau levels and with taking into account all Landau levels are plotted with different line styles.

As we see, even taking into account the mixing between two Landau levels changes the dispersions considerably (see the differences between solid and short dash lines in Figure 3). However, the calculations with mixing between three Landau levels (long dash lines) are already close to the exact results (dotted lines), except for the high-lying magnetoplasmon modes. It is also seen, that the mixing considerably changes the dispersions even at moderate *r*_s _(see, e.g., Figure 3d at *r*_s _= 0.5). Note that the mixing usually decreases magnetoplasmon energies and does not affect the long-wavelength linear asymptotics of their dispersions.

Therefore, we conclude here that convergence of magnetoplasmon dispersions in rather fast upon increasing a number of Landau levels taken into account. Several lowest Landau levels are sufficient to obtain rather accurate results. On the other hand, calculations in the lowest Landau level approximation, i.e., without taking into account the mixing, can give inaccurate results, especially in a region of intermediate momenta $q\sim l_{H}^{-1}$.

## 5 Conclusions

We studied influence of Landau level mixing in graphene in quantizing magnetic field on properties of elementary excitations—magnetoexcitons and magnetoplasmons—in this system. Virtual transitions between Landau levels, caused by Coulomb interaction, can change dispersions of the excitations in comparison with the lowest Landau level approximation.

Strength of Coulomb interaction and thus a degree of Landau level mixing can be characterized by dimensionless parameter *r*_s_, dependent in the case of graphene only on dielectric permittivity of surrounding medium. By embedding graphene in different environments, one can change *r*_s _from small values to *r*_s _≈ 2 [49].

We calculated dispersions of magnetoexcitons in graphene and showed that the mixing even between few Landau levels can change the dispersion curves significantly at *r*_s _> 1. However, at small *r*_s _the role of the mixing is negligible, in agreement with the other works [20,25]. Then the question about convergency of such calculations upon increasing a number of involved Landau levels have been raised.

We performed calculations of magnetoexciton energies at rest with taking into account stepwise increasing number of Landau levels and found their inverse-square-root asymptotics. By evaluating limiting values of these asymptotics, we calculated magnetoexciton energies with infinite number of Landau levels taken into account. We demonstrated that influence of remote Landau levels of magnetoexciton energies is strong, especially at large *r*_s_. Also it was found that calculations with taking into account even several Landau levels provide results, rather far from exact ones.

Also dispersion relations of magnetoplasmons in graphene were calculated in the random phase approximation with taking into account different numbers of Landau levels. We showed that even few Landau levels for electron and hole are sufficient do obtain accurate results, however the lowest Landau level approximation (i.e., calculations without taking into account the mixing) provide inaccurate results, especially for intermediate momenta and high-lying magnetoplasmon modes.

In our article, we focused on the role of Coulomb interaction only in the electron-hole channel. Another many-body mechanism, affecting observed magnetoexciton energies, is renormalization of single-particle energies due to exchange with filled Landau levels in the valence band of graphene, which was considered elsewhere [20,21,24,30,40]. An important result of our study is that breakdown of the Kohn theorem in graphene leads to strong corrections of magnetoexciton energies not only due to exchange self-energies, but also due to virtual transitions caused by Coulomb interaction between electron and hole. One can distinguish these two contributions in experiments by measuring full dispersion dependencies (at nonzero momenta) of spatially indirect magnetoexcitons formed by electrons and holes in parallel graphene layers by means of registration of luminescent photons in additional parallel magnetic field (similarly to the experiments with semiconductor quantum wells [50]).

We considered magnetoexcitons in the ladder approximation and magnetoplasmons in the random phase approximations without taking into account vertex corrections and screening. Estimating the role of these factors, especially in the strong-interacting regime at large *r*_s_, is a difficult task and will be postponed for future studies.

The results obtained in our study should be relevant for magneto-optical spectroscopy of graphene [28,29,31,51-53] and for the problem of Bose-condensation of magnetoexcitons [54-56]. Excitonic lines in optical absorption or Raman spectra of graphene can give experimental information about energies of elementary excitations. Magnetoexcitons and magnetoplasmons can be observed also as constituents of various hybrid modes—polaritons [57], trions [58], Bernstein modes [59] or magnetophonon resonances [60].

## Competing interests

The authors declare that they have no competing interests.

## Authors' contributions

YEL formulated the problem, provided the consultations on key points of the work and helped to finalize the manuscript. AAS carried out the calculations and wrote the manuscript draft. Both authors read and approved the final manuscript.
